# Communicating with patients and families about illness progression and end of life: a review of studies using direct observation of clinical practice

**DOI:** 10.1186/s12904-021-00876-2

**Published:** 2021-12-08

**Authors:** Stuart Ekberg, Ruth Parry, Victoria Land, Katie Ekberg, Marco Pino, Charles Antaki, Laura Jenkins, Becky Whittaker

**Affiliations:** 1 School of Psychology & Counselling, Queensland University of Technology, Victoria Park Road, Kelvin Grove, Queensland 4059 Australia; 2 Centre for Healthcare Transformation, Queensland University of Technology, Brisbane, Queensland Australia; 3 School of Social Sciences and Humanities, Loughborough University, Brockington Building, Epinal Way, Margaret Keay Road, Loughborough, LE11 3TU UK; 4 School of Early Childhood & Inclusive Education, Queensland University of Technology, Victoria Park Road, Kelvin Grove, Queensland 4059 Australia; 5 School of Health & Rehabilitation Sciences, University of Queensland, St Lucia, Queensland 4072 Australia

**Keywords:** Communication, Difficult conversations, Serious news, Bad news, Conversation analysis, Discourse analysis, Rapid review, Palliative care

## Abstract

**Background:**

There is growing recognition that a diverse range of healthcare professionals need competence in palliative approaches to care. Effective communication is a core component of such practice. This article informs evidence-based communication about illness progression and end of life through a rapid review of studies that directly observe how experienced clinicians manage such discussions.

**Methods:**

The current rapid review updates findings of a 2014 systematic review, focussing more specifically on evidence related to illness progression and end-of-life conversations. Literature searches were conducted in nine bibliographic databases. Studies using conversation analysis or discourse analysis to examine recordings of actual conversations about illness progression or end of life were eligible for inclusion in the review. An aggregative approach was used to synthesise the findings of included studies.

**Results:**

Following screening, 26 sources were deemed to meet eligibility criteria. Synthesis of study findings identified the structure and functioning of ten communication practices used in discussions about illness progression and end-of-life.

**Conclusion:**

The ten practices identified underpin five evidence-based recommendations for communicating with patients or family members about illness progression and end of life.

**Supplementary Information:**

The online version contains supplementary material available at 10.1186/s12904-021-00876-2.

## Background

Specialists in palliative care recognise that other healthcare professionals need support to feel confident and able to deliver high quality care to people with life-threatening and life-limiting illnesses, encompassing diagnosis to end of life [1, 2]. The importance of generalist palliative care has become particularly prominent during the Coronavirus Disease 2019 (COVID-19) pandemic [3, 4], which has highlighted that specialist palliative care alone cannot provide the comprehensive palliative care required by all patients. Instead, clinicians who are unfamiliar with palliative care skills, including specialists who do not routinely provide palliative care as well as practitioners at an early stage of training, will encounter situations where they are required to deliver such care [4–6]. This care can include managing discussions about illness progression and, in some cases, end of life [4]. There are known benefits to these conversations [7–10], but it is also known that many clinicians find discussions about prognosis and dying deeply challenging [11]. During high-pressure periods such as the COVID-19 pandemic, the challenging nature of these conversations can be intensified, especially for clinicians who do not routinely manage such discussions outside these high-pressure periods [5, 12, 13].

There is clearly scope to improve skills and confidence among generalists. Specialist palliative care clinicians are well placed to support their frontline colleagues [14, 15]. In addition to using their own professional experience and expert opinion, skilled practitioners should take advantage of research highlighting practices that can be used for communicating with and about patients at end of life [16]. This rapid review is designed to facilitate this, updating a previous review [16] by synthesising high-quality evidence that identifies how experienced clinicians manage discussions about illness progression and end of life.

### Direct real-life evidence

High-quality evidence about clinical communication is achieved through studies that directly examine video or audio recordings of real-life clinical practice [17]. This approach avoids the limitations of self-report methods, which can only provide indirect and partial evidence of the structure, function, and outcomes of communication in real-life consultations [18, 19]. ‘Gold standard’ approaches to the study of recorded clinical communication are conversation analysis and discourse analysis [17, 20, 21]. In contrast to alternative approaches, such as deductively pre-specified coding systems, which sacrifice detail and specificity to achieve generalisability [21], conversation analysis and discourse analysis employ detailed and inductive methods to understand how specific communication practices function in particular contexts, while also identifying communication practices that can be used across contexts [21–23].

Recent decades have seen development of a cumulative body of evidence generated by studies that directly examine conversations about potentially sensitive matters such as illness progression and end of life [19]. This evidence enabled a systematic review of conversation analysis and discourse analysis studies, published in 2014, which provides guidance on how to communicate with patients and their families about sensitive future matters [16]. Further growth in evidence has occurred since then [24]. The impetus for this current paper is this increase in research evidence, as well as the increased frequency of discussions about illness progression and end of life during the COVID-19 pandemic. This paper reports a rapid review that updates findings of the 2014 systematic review, and focuses more tightly upon evidence related to illness progression and end-of-life conversations.

## Methods

### Rapid review approach

Rapid reviews are a restricted type of systematic review in which the review process is simplified with the aim of increasing efficiency [25, 26]. Using accelerated or streamlined methods reduces the time it takes to report findings and develop guidelines, while minimally impacting quality [25, 27]. Crucially, rapid reviews involve a close relationship between the review team, the end-user, and the needs of decision-makers, driven by matters such as clinical urgency and limited time resources [25, 28, 29]. A global health crisis, such as the COVID-19 pandemic, elevates the need for up-to-date syntheses of important evidence [30].

Rapid reviews limit their scope in a variety of ways, including narrowing the scale of the research questions, constraining the number of databases searched, and restricting the data extraction [25]. The decision as to which items are streamlined requires careful consideration [31]. Rapid review quality is increased by ensuring that the review team includes members with experience in the design and conduct of full systematic reviews [25, 26], and transparent reporting of the methods that were used in a review [25].

This rapid review was informed by guidelines developed specifically for systematically reviewing and synthesising evidence from conversation analytic and discourse analytic research [17]. At the time the review was conducted, although interim guidance was available for rapid reviews to develop scientific briefs [32], consensus guidelines for using rapid reviews to develop practical guidance were unavailable [33, 34]. Additional guidance was published while the review was underway [35], and has been incorporated where feasible. Where rapid reviewing guidelines conflicted with specialist guidelines for systematically review conversation analytic and discourse analytic research [17], the latter were followed. Following suggestions in published research literature [35–37], common systematic review methods were adapted for this rapid review. The following adaptations were made: 1) not publishing a protocol before commencing; 2) using rapid review to update a previous systematic review [16]; 3) excluding ‘grey literature’; 4) using one reviewer to screen search results to identify sources meeting eligibility criteria, with a second reviewer used to screen at least 20% of manuscripts; 5) not screening the reference lists of included studies to identify additional sources; 6) dividing critical appraisal and data extraction work among members of the review team; and 7) having only one reviewer undertake critical appraisal and data extraction from included studies.

### Eligibility criteria

The aim for search strategy underpinning the current review was to add studies published since the 2014 systematic review [16]. The focus of the current review was more specific than the 2014 review, which focused on discussions about difficult future matters. Growth in research evidence since 2014 enabled the current review to focus more narrowly on discussions between healthcare users and professionals that relate specifically to illness progression or end of life. All sources from the 2014 systematic review were considered for inclusion in the current rapid review, although many were not expected to meet the more focused eligibility criteria for the current review. Only studies that included direct evidence of communication about end of life or illness progression of a life-limiting illness, in the form of audio or audio-visual recordings of actual (i.e., ‘real life’) conversations, were eligible for inclusion. As noted, conversation analysis and discourse analysis are leading approaches to studying these types of data. The review was therefore restricted to studies employing only these approaches. Studies where the bulk of analysis involved use of coding frameworks were excluded [17]. Peer reviewed journal articles and published monographs and book chapters were considered for inclusion. Only studies published in English that examined conversations conducted in English-speaking countries (e.g., the United Kingdom and the United States of America) were eligible for inclusion.

### Search strategy

The search strategy employed for the 2014 systematic review [16, 17] was adapted for this rapid review. After initial piloting, one search term (‘future’) was removed to expand the scope of the search and incorporate a greater range of published research. The same bibliographic databases used in the 2014 systematic review were searched for this rapid review: MEDLINE, EMBASE, CINAHL, PsycINFO, Web of Science, and Scopus were searched by one reviewer (SE), and Sociological Abstracts, ASSIA and Amed by another (VL). A third reviewer (LJ) independently screened 20% of the search results to enable a check of consistency in extraction against the inclusion criteria. The search strategy for MEDLINE is available as Supplementary File 1.

Searches were restricted to research published following 1 May 2014, which was the day following the end of the search range used for the previous review [16]. The final search was conducted on 8 December 2020.

### Study selection

Search results were initially screened by title; where necessary, the abstract or full text were screened to determine whether the study met the eligibility criteria.

### Study appraisal and data extraction

Quality appraisal is not suitable for the types of studies included in this review [17]. A data extraction form, developed by some of the co-authors [17], was simplified based on information reported in their 2014 systematic review [16]. In addition to key information about each study, all fragments of data (i.e., transcripts of real-life conversations) published within the study were extracted. Appraisal and data extraction were conducted simultaneously, to facilitate rapid review. Included studies were divided among a team of reviewers (SE, VL, KE, MP, CA) to expedite this process. A second reviewer from a team of reviewers (SE, RP, VL, MP, CA) checked each study for correctness and completeness of extracted data.

### Data synthesis

The same aggregative approach employed in the 2014 review [16] was also used for the current review. This review process involved procedures used in primary conversation and discourse analysis research: detailed case-by-case analysis, proceeding to analytic generalisations across cases, while ensuring any such generalisations remain congruent with the details of individual cases [38, 39]. Because generalisations in these types of research relate to phenomena not populations [39], this review focuses on the function of communication practices. Nevertheless, to enable exploration of the transferability of findings [39], including across different populations and clinical contexts, only findings made in more than two included studies were included in the synthesis. The focus of synthesis was restricted to analytic claims made by the original study authors, rather than those that might be additionally identified by the review team through the pooling of data from across the included studies. Aggregation was led by one reviewer (SE), with critical input from each review team member. Deliberation among the team continued until consensus was reached.

## Results

**Fig. 1:**
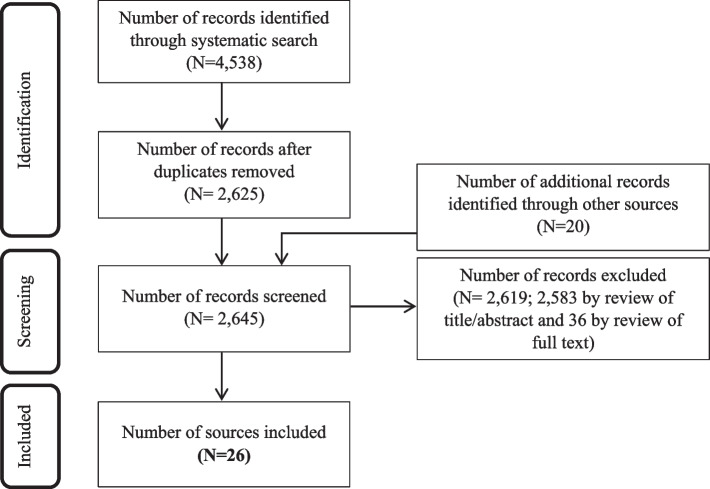
Screening results

As shown in Fig. 1, 2625 unique sources were identified through electronic searching of literature published between 1 May 2014 and 8 December 2020. In addition, 19 sources from the 2014 review [16] were included, along with one additional source identified independently by a review team member, based on their knowledge of the literature.

Following screening, 26 studies were deemed to meet eligibility criteria. These studies examined discussions between healthcare users and professionals that relate to end of life and occurred across a variety of clinical settings: hospice or palliative care [40–48], oncology [49–56], intensive care [57, 58], cardiology [59], counselling [60–63], and therapy [64, 65]. Each of these studies, including those from counselling and therapy sessions, were included because they related to patients with life-threatening or life-limiting conditions and involved discussions about illness progression and end of life. The included studies examined patients with different types of life-threatening and life-limiting conditions, patients at different stages of an illness trajectory, and patients of different ages (including children). Further details about the included studies are available in Supplementary File 2.

Data synthesis identified ten communication practices, which are each described in the below subsections. Given this focus of this review on providing evidence to guide clinical practice, only practices that are used by clinicians are reported, although some practices were used by both clinicians and patients or family members (e.g., referring to the future indirectly). The support for each communication practice is reported in Table 1. On the basis of evidence about these practices, five core evidence-based recommendations have been produced, and are reported in tables alongside their supporting evidence. One of the included studies was not included in the synthesis [58], as its findings were distinct from the other included studies.

**Table 1:** Sources supporting the use of particular communication practices

Practice	Number of sources	Settings where the practice has been observed
Providing opportunities for patients or family members to raise illness progression or end of life matters to discuss	5	Palliative care/hospice [40, 41] Counselling [60, 61] Oncology [49]
Seeking a patient or family member’s perspective about a specific illness progression or end of life matter	5	Palliative care/hospice [41, 42] Intensive care [57] Oncology [49] Therapy [64]
Referring to the future indirectly	9	Oncology [49–53] Palliative care/hospice [42–44] Cardiology [59]
Referring to the future directly	7	Oncology [50, 51, 54, 55] Palliative care/hospice [42–44]
Linking previous discussions or events relating to illness progression or end of life	8	Palliative care/hospice [40, 42, 45, 46] Counselling/therapy [60–62] Therapy [65]
Using hypothetical scenarios to foster discussions about illness progression or end of life	7	Counselling [60–63] Hospice [46] Oncology [49] Cardiology [59]
Framing illness progression and end of life in general terms	5	Oncology [50, 51] Counselling [60, 63] Hospice [46]
Acknowledging uncertainty about the future	2	Hospice [44] Oncology [52]
Displaying sensitivity	6	Counselling [60, 62, 63] Palliative care/hospice [43, 47] Oncology [49]
Emphasising the positive	5	Oncology consultations [49, 52, 56] Palliative care/hospice [40, 48]

### Providing opportunities for patients or family members to raise illness progression or end of life matters to discuss

Some of the included studies identify ways clinicians provide opportunities for patients or family members themselves to nominate matters they would like to discuss during a consultation. Often used towards the beginning of consultations, when the agenda for the consultation is being set, such practices have also be found to be used at subsequent points in the consultation, where they provide further opportunities for a patient or family member to raise additional matters they would like to discuss [66]. Commonly, such opportunities are provided through open questions, such as *“Is there anything else you guys wanted to mention or?“* [40]. In this instance, the question does not specify a possible topic, and the inclusion of “or” at the end of the question orients to a the possibility of a disconfirming response not being problematic [67]. The open-ended design of such questions provides space for a patient or family member to mention matters they might like to discuss, but without narrowing the focus to matters relating to illness progression or end of life [16]. Although providing these opportunities does not guarantee a patient or family member will raise illness progression or end of life, there is evidence that patients sometimes do take the opportunity to raise these matters [40, 41]. This evidence provides the first part of the support for Recommendation 1: Ascertain a patient or family member’s perspective before offering your own (see Table 2). Additional evidence comes from studies of the next practice.

**Table 2:** Recommendation 1: Ascertain a patient or family member’s perspective before offering your own

**Try to find out what a patient or family member would like to get out of a conversation**	
Where possible, create opportunities for patients or family members to raise matters they would like to discuss. They may indicate their readiness to discuss illness progression or end of life. If they do not, it may nonetheless be possible to get a sense of how open, or reluctant, they might be to engage with such matters. What you say next can be informed by this.	
**Try to find out a patient’s or family member’s perspective about the future**	
Before offering your own perspective about a patient’s future, try to ascertain a patient’s or family member’s perspective about this matter. This will help you to take that perspective into account when deciding how to offer your own perspective and when deciding when, how, and whether to encourage and pursue their engagement with matters relating to illness progression and end of life.	

### Seeking a patient or family member’s perspective about a specific illness progression or end of life matter

In addition to generic attempts to provide opportunities in which patients or family members can raise illness progression or end of life matters to discuss, in some of the included studies clinicians were observed to seek a patient or family member’s perspective about a more specific matter. These solicitations are often achieved through a ‘perspective display invitation’, which seeks another person’s opinion [68]. Examples include: *“Do you know her preferences of the kind of quality of life she would want?“* [57] and *“What do you see as (pause) as the- happening in the future?“* [49] Patients and family members sometimes responded to these types of questions by raising matters related to illness progression or end of life [64]. Research evidence has also documented how, by first soliciting a patient or family member’s perspective, the clinician then incorporates the patient’s or family member’s perspective in what they go on to say, even if their perspective diverges from that of the patient or family member [42, 68]. This evidence provides the second part of the support for Recommendation 1: Ascertain a patient or family member’s perspective before offering your own (see Table 2).

### Referring to the future indirectly

Many of the included studies identify a diverse range of practices people use to more or less indirectly refer to illness progression and end of life. The most indirect practices included clinicians alluding to the possibility of illness progression and end of life, such as by stating that current treatment has been exhausted*: “I think we’ve gotten as much as we’re going to get from this treatment”* [53], or through allusive references to the future: *“so coming back to what you were saying before…part of it is the fear of what might happen?“* [42] The most direct practices, that nevertheless avoid completely explicit references such as ‘death’ or ‘dying’, are euphemisms such as *“when he passes.”* [43] Towards the middle of this spectrum of indirect practices are references to time, such as *“I think he probably has a limited amount of time now.”* [44] There is some evidence that indirect discussions about illness progression and end of life are the default way that patients, family members, and clinicians talk about such matters. This is particularly the case when the patient is either involved in the conversation or is a significant figure for one or more parties to the conversation [41–43, 49].

Some studies highlight challenges associated with indirect references to the future. For example, when a clinician’s statement that current treatment has been exhausted is followed by descriptions of what can be done, the focus of discussion tends to remain on active treatment rather than palliative approaches to care [53]. This is considered further below, in relation to emphasising the positive. In contrast to studies that identify challenges associated with indirect references to the future, other studies identify ways indirect references about the future provide opportunities to talk about illness progression and end of life. For example, clinicians can monitor what patients and family members say, identify comments that may relate to end-of-life considerations, and solicit elaborations on these. A question already considered above is one such instance: *“so coming back to what you were saying before…part of it is the fear of what might happen?“* [42]. This question functions as an elaboration solicitation, creating space where the patient subsequently discussed end of life. Importantly, the clinician created this space for end of life talk without referring to this future outcome directly. Such instances where clinicians used indirect references to the future to promote talk about illness progression and end of life provide the first part of the support for Recommendation 2: Mirror the language of the patient or family (see Table 3). There is, however, additional evidence that comes from studies of the next practice, which qualifies the extent to which clinicians should mirror the language of the patient or family member.

**Table 3:** Recommendation 2: Where possible, mirror the language of the patient or family

**If a patient or family member discusses the future indirectly or allusively, try to do the same but without increasing ambiguity or concealing fateful outcomes**	
In many societies, it is common for dying and death to be discussed indirectly. If patients or family members talk about the future indirectly and this does not appear to create the possibility for misunderstanding or ambiguity, in particular about fateful outcomes, try to use similar language. As you talk to them, they may come to discuss the future more directly, in which case you can adjust your language accordingly. When there are important reasons to talk about the future, despite a patient or family member not displaying willingness to do so, Recommendation 3 provides ways to facilitate this.	

### Referring to the future directly

In contrast to practices that discuss future deterioration and end of life indirectly, practices where such matters are referred to directly have also been observed and analysed in many of the included studies. Evidence suggests discussions about end of life are sometimes initiated by clinicians indirectly, and subsequently made explicit by patients [42, 49]. In general, clinicians tend to refer directly to end of life only after patients themselves have made a direct reference to this [50, 51].

There are exceptions, however, where clinicians initiate more direct discussions about illness progression and end of life. Sometimes, in instances where patients or family members have not taken up prior opportunities to talk about illness progression and end of life, clinicians can respond by referring to these matters more directly [42, 49], such as with: *“Do you worry about what’s coming?“* [42]. Together with the evidence about indirectness presented above, this evidence about direct communication underpins Recommendation 2: Where possible, mirror the language of the patient or family (see Table 3).

### Linking previous discussions or events relating to illness progression or end of life

Many of the included studies found that clinicians can mention something said or done in the recent or distant past that is related to illness progression and end of life, then use this to promote further discussion about these matters. Examples include: *“So coming back to what you were saying before…part of it is the fear of what might happen?“* [42]; *“Do you remember when you first came on the ward here?...Things were pretty desperate…And we got you on a little syringe pump with the pain medicine in?“* [46]. There are documented instances where patients respond to such solicitations with matters relating to illness progression and end of life [42]. Clinicians can also link to something a patient has not said, to provide a basis for asking about it: *“You haven’t mentioned AIDS as a concern today. How much of a concern is that?“* [61]. This evidence provides the first part of the support for Recommendation 3: Create opportunities to discuss the future (see Table 4). Additional evidence comes from studies of the following two practices.

**Table 4:** Recommendation 3: Create opportunities to discuss the future

The following strategies are particularly useful for occasions where patients or family members seem reluctant to engage in discussions about future illness progression or end of life.	
**Highlight connections between what a patient or family member has said and what you are saying now**	
To promote further talk about future illness progression and end of life, try bringing up something the patient or family member has mentioned before about the future, then use this to promote further discussion about this matter. You can help them link concerns they have already expressed with concerns about and plans for end of life.	
**Use hypothetical scenarios to explore possibilities when you think it is important to talk about the future in this conversation**	
Talking about the future hypothetically means patients and family members do not need to agree that this is necessarily how their future will transpire. Evidence suggests people can be more open to engaging in these types of hypothetical discussions. If you judge it important to pursue discussion about a patient's illness progression and end of life, hypothetical scenarios can be used to promote this.	
**Refer to illness progression and end of life generally, if you are unsure how a patient or family member will react**	
Mentioning something in relation to people generally, rather than the patient specifically (e.g., “when people are very ill…”), can be useful when you want to raise something that a patient or family member hasn’t already hinted at, or where you want to provide them with an opportunity to recognise its relevance to them and apply it to their own situation, but without forcing them to do so.	

### Using hypothetical scenarios to foster discussions about illness progression or end of life

Many of the included studies examined how hypothetical future scenarios are used to foster discussion about matters relating to illness progression and end of life [16]. Examples include: *“And if there was a bit of uh bang, if there was a bit’v bleeding or some other crisis, how would you want to handle that do you think?“* [46]; *“If you- supposing- I mean this is just supposing, supposing you had got infected or were to get infected…“* [60] There are contexts where these practices appear to be particularly effective at promoting discussion about illness progression and end of life. These contexts include circumstances where a patient or family member has displayed reticence to discuss these matters, or to question a patient or family member’s expressed plans or expectations for the future [16, 46]. This evidence provides the second part of the support for Recommendation 3: Create opportunities to discuss the future (see Table 4). Additional evidence comes from studies of the next practice.

### Framing illness progression and end of life in general terms

In contrast to hypothetical scenarios, which involve discussions related to the individual patient, several studies included in the review identify another practice which involves framing matters abstractly, as something that could be faced by people more generally rather than a particular patient specifically [16]. This generalised framing occurs in the following instance: *“…sometimes when people are really unwell…what we do is we get them some medicine at home.”* [46]. There is some evidence that generalised statements are more likely to be used in relation to matters that have not been raised by a patient or family member in the past [60]. Their use softens the direct relevance of the matter being discussed in relation to the patient [46]. This evidence provides the third part of the support for Recommendation 3: Create opportunities to discuss the future (see Table 4).

### Acknowledging uncertainty about the future

A few of the included studies examined a practice that involves clinicians using expressions that qualifies their level of certainty, as well as explicit statements of uncertainty. The first part of the following instance includes qualifying expressions (‘looks like’ and ‘probably’), and the second part an explicit statement of uncertainty: *“This looks like the last days probably…We have learned that we have no idea to predict how many.”* [44]. This practice demonstrates that prognostic uncertainty does not need to prevent discussions about illness progression and end of life [44]. This evidence provides the support for Recommendation 4: Be clear about uncertainty (see Table 5).

**Table 5:** Recommendation 4: Be clear about uncertainty

**Acknowledge the components of the patient’s future that are uncertain**	
Even when illness progression and end of life are certain, explain things that are less certain, such as the timeframe for progression.	

### Displaying sensitivity

In addition to practices described above, such as discussing illness progression and end of life indirectly, several of the included studies identify other practices with which clinicians can empathise with a patient or family member’s situation during discussions of illness progression and end of life. Such communication practices include explicit expressions of sensitivity, such as a clinician’s claim to understand a patient’s emotional experience: *“I know it’s not always the easiest thing to uh to chat about.”* [47]. Evidence also suggests silence or brief responses such as ‘mm’ can be effective following the initiation of potentially sensitive matters such as illness progression and end of life [63]. Two of the included studies indicate that talk about such matters is more likely to contain hesitations, delays, cut-off words, and repeated words or phrases [43, 62]. As noted in the 2014 review [16], there is limited research considering how non-verbal behaviour, such as touch, can be used to convey sensitivity. This evidence provides the first part of the support for Recommendation 5: Display sensitivity (see Table 6). Additional evidence comes from studies of the next practice.

**Table 6:** Recommendation 5: Display sensitivity

**Use verbal and non-verbal displays of sensitivity**	
There are many ways you might display sensitivity when discussing illness progression and end of life. Explicit statements can acknowledge the difficulties around talking about these delicate matters. Once the topic has been raised, allowing periods of silence or brief responses such as ‘mm’ can encourage a patient to say more.	
**Acknowledge positives, but not too soon**	
A further way to display sensitivity to emotional distress is to acknowledge positives, but delay doing so until it is time to close this part of the conversation.	

### Emphasising the positive

Some of the included studies consider ways that discussions about illness progression and end of life routinely culminate in shifts to discussing something positive, by clinicians, patients and family members. This occurs in the second sentence of the following example: *“Essentially erm the cancer’s sort’ve overwhelming the body and the heart and other vital organs can’t cope anymore. And then our focus now is very much on keeping Simon as comfortable as possible.”* [48]. As noted in the 2014 review, such practices can be used to sustain hope and preserve relationships, but they can also divert the conversation, thereby preventing further talk about illness progression and end of life [16]. The effect of emphasising the positive can make this a useful practice for closing down prognostic talk after this has been discussed adequately [48, 53]. This evidence provides the second part of the support for Recommendation 5: Display sensitivity (see Table 6).

## Discussion

This rapid review has synthesised findings from studies examining conversations about illness progression and end of life. The review focuses on evidence from direct observational studies of real-life conversations to understand how specific communication practices function in particular contexts [17, 20, 21]. Since publication of a systematic review on this topic in 2014 [16], evidence in this area has expanded, in particular in the specialist areas of palliative and hospice care. The synthesis of findings from currently available evidence underpins five core recommendations for clinicians who need to discuss illness progression and end of life with patients or their families (see Tables 2, 3, 4, 5 and 6).

During the Coronavirus Disease 2019 (COVID-19) pandemic, professionals who do not routinely provide critical or end of life care have been thrust into situations where they have needed to discuss difficult matters with patients or their families. Even experienced palliative care clinicians are also facing fresh challenges [5, 12]. Notwithstanding these challenges, studies of communication demonstrate that across a wide variety of contexts from courtrooms to classrooms, medical interactions to board meetings, professionals do not constantly invent entirely bespoke systems of communication, but instead adjust everyday communication practices to fit their institutional tasks and roles [69, 70]. In the same way, while the novel clinical character of COVID-19 and related circumstances and communication needs are striking [5, 12], it is important to recognise that rather than adopting an entirely new set of practices for difficult conversations, people most commonly adapt existing resources. For this reason, the current review considered studies that examined different clinical settings, patients with different types of life-threatening and life-limiting illness, patients at different stages of an illness trajectory, and patients of different ages (including children). This scope enabled identification of communication practices that may be transferrable across diverse aspects of clinical practice. The recommendations made in this article equip both experienced clinicians and those new to managing difficult conversations with practices that are known to help manage conversations about illness progression and end of life, which are likely to be transferrable to challenging circumstances such as those during the COVID-19 pandemic.

The evidence-based recommendations listed across Tables 2, 3, 4, 5, and 6 are not prescriptive, nor do they recommend clinicians use scripted phrases. This approach recognises that the contingencies of communication mean these social encounters can rarely – if ever – ‘follow the script’. [71] The recommendations reflect this complexity, to help explain, for instance, why people discuss sensitive future matters indirectly in some circumstances and explicitly in other circumstances (see Table 3). The communication practices described across Tables 2, 3, 4, 5 and 6 range from some that are relatively more cautious and indirect, to those that are relatively more direct and strongly encourage discussions about illness progression and end of life. It is important to consider, on a case-by-case basis, which approaches are likely to be most suitable. As always in evidence-based practice, quality evidence should inform, but not replace, clinicians’ decisions about how to provide care that is appropriate for individual patients and their circumstances [72].

There are already many strategies and frameworks designed to inform the conduct of discussions about illness progression and end of life [73, 74]. Prominent contemporary approaches include the SPIKES protocol [75], VitalTalk [76], and the Serious Illness Conversation Guide (SICG) [77]. The recommendations made across Tables 2, 3, 4, 5 and 6 have important similarities and differences to these resources. For instance, recommendations to elicit the patient’s perspective (SPIKES), assess perception of illness (VitalTalk), and assess illness understanding (SICG) are consistent with the second practice underpinning the evidence-based recommendation made in Table 2. This review also documents practices that extend beyond those recommended in these available resources, such as considering why specific communication practices such as communicating indirectly (Table 3) and using hypothetical scenarios (Table 4) may be useful. This highlights a key advantage of using direct empirical evidence to understand what constitutes effective communication in clinical settings [78]. Such an approach, which is exemplified by conversation analysis and discourse analysis methods, highlights ways experienced clinicians adapt their communication practice in response to the contingencies of communication in real-life clinical practice. It is likely that such expertise can only ever be partially captured by communication frameworks.

Considerable progress has been made in developing high-quality evidence to inform conversations about illness progression and end of life. ‘Gold standard’ research based on direct and detailed analysis of audio- or video-recorded real-life discussions about illness progression and end of life has substantially increased since a systematic review was published in 2014 [16]. Nevertheless, because this review is based on inductive methods of generating knowledge, the understandings of clinical communication that this affords is partial. Further research is therefore likely to yield additional insights into the nature of conversations about illness progression and end of life. In particular, further research is needed to understand ways clinicians explore options with patients and families, as this was only considered in one of the included studies [58].

## Conclusion

There is urgent need for capacity among a broad range of healthcare professionals to adopt palliative approaches to care [1, 2]. The COVID-19 pandemic has highlighted this need. This rapid review synthesises direct evidence of ways experienced clinicians manage challenging discussions about illness progression and end of life. The identification of common types of communicative practices used across these diverse clinical settings increases confidence that the findings of this review are transferrable to the discussions about illness progression and end of life that clinicians may need to have across a variety of clinical settings. Through the accumulation of detailed analysis of such conversations, increasingly clear evidence has emerged to inform this poignant part of clinical practice. There is now scope to use this evidence to improve the quality, safety, and experience of healthcare.

## Supplementary Information

**Additional file 1: Supplementary file 1**: Example search strategy (MEDLINE).

**Additional file 2: Supplementary file 2**: Characteristics of included studies.

## Data Availability

All data analysed are included in published literature that were identified through the following bibliographic databases: MEDLINE, EMBASE, CINAHL, PsycINFO, Web of Science, Scopus, Sociological Abstracts, ASSIA and Amed. For further information about the availability of these data, please contact the corresponding author.
