# Supplementary material for: Communicating with patients and families about illness progression and end of life: a review of studies using direct observation of clinical practice
Source: BMC Palliat Care. 2021 Dec 8;20:186. doi: 10.1186/s12904-021-00876-2 (PMC8651503; doi:10.1186/s12904-021-00876-2)
Supplement: Supplementary file 1 — Additional file 1: Supplementary file 1: Example search strategy (MEDLINE). [file 12904_2021_876_MOESM1_ESM.docx]

**Supplementary file 1: Example search strategy (MEDLINE)**

| **#** | **Query** | **Limiters/Expanders** | **Run via** |
| --- | --- | --- | --- |
| S17 | S3 AND S10 AND S16 | Limiters - Date of Publication: 20140501-20201208 | Interface - EBSCOhost Research Databases |
|  |  | Expanders - Apply equivalent subjects | Search Screen - Advanced Search |
|  |  | Search modes - Boolean/Phrase | Database - MEDLINE |
| S16 | S11 OR S12 OR S13 OR S14 OR S15 | Expanders - Apply equivalent subjects | Interface - EBSCOhost Research Databases |
|  |  | Search modes - Boolean/Phrase | Search Screen - Advanced Search |
|  |  |  | Database - MEDLINE |
| S15 | troubles | Expanders - Apply equivalent subjects | Interface - EBSCOhost Research Databases |
|  |  | Search modes - Boolean/Phrase | Search Screen - Advanced Search |
|  |  |  | Database - MEDLINE |
| S14 | decision* | Expanders - Apply equivalent subjects | Interface - EBSCOhost Research Databases |
|  |  | Search modes - Boolean/Phrase | Search Screen - Advanced Search |
|  |  |  | Database - MEDLINE |
| S13 | end-of-life | Expanders - Apply equivalent subjects | Interface - EBSCOhost Research Databases |
|  |  | Search modes - Boolean/Phrase | Search Screen - Advanced Search |
|  |  |  | Database - MEDLINE |
| S12 | palliative | Expanders - Apply equivalent subjects | Interface - EBSCOhost Research Databases |
|  |  | Search modes - Boolean/Phrase | Search Screen - Advanced Search |
|  |  |  | Database - MEDLINE |
| S11 | prognos* | Expanders - Apply equivalent subjects | Interface - EBSCOhost Research Databases |
|  |  | Search modes - Boolean/Phrase | Search Screen - Advanced Search |
|  |  |  | Database - MEDLINE |
| S10 | S4 OR S5 OR S6 OR S7 OR S8 OR S9 | Expanders - Apply equivalent subjects | Interface - EBSCOhost Research Databases |
|  |  | Search modes - Boolean/Phrase | Search Screen - Advanced Search |
|  |  |  | Database - MEDLINE |
| S9 | linguistic* | Expanders - Apply equivalent subjects | Interface - EBSCOhost Research Databases |
|  |  | Search modes - Boolean/Phrase | Search Screen - Advanced Search |
|  |  |  | Database - MEDLINE |
| S8 | sequential analysis | Expanders - Apply equivalent subjects | Interface - EBSCOhost Research Databases |
|  |  | Search modes - Boolean/Phrase | Search Screen - Advanced Search |
|  |  |  | Database - MEDLINE |
| S7 | conversation analysis | Expanders - Apply equivalent subjects | Interface - EBSCOhost Research Databases |
|  |  | Search modes - Boolean/Phrase | Search Screen - Advanced Search |
|  |  |  | Database - MEDLINE |
| S6 | discourse analysis | Expanders - Apply equivalent subjects | Interface - EBSCOhost Research Databases |
|  |  | Search modes - Boolean/Phrase | Search Screen - Advanced Search |
|  |  |  | Database - MEDLINE |
| S5 | video* | Expanders - Apply equivalent subjects | Interface - EBSCOhost Research Databases |
|  |  | Search modes - Boolean/Phrase | Search Screen - Advanced Search |
|  |  |  | Database - MEDLINE |
| S4 | audio* | Expanders - Apply equivalent subjects | Interface - EBSCOhost Research Databases |
|  |  | Search modes - Boolean/Phrase | Search Screen - Advanced Search |
|  |  |  | Database - MEDLINE |
| S3 | S1 OR S2 | Expanders - Apply equivalent subjects | Interface - EBSCOhost Research Databases |
|  |  | Search modes - Boolean/Phrase | Search Screen - Advanced Search |
|  |  |  | Database - MEDLINE |
| S2 | interact* | Expanders - Apply equivalent subjects | Interface - EBSCOhost Research Databases |
|  |  | Search modes - Boolean/Phrase | Search Screen - Advanced Search |
|  |  |  | Database - MEDLINE |
| S1 | communicat* | Expanders - Apply equivalent subjects | Interface - EBSCOhost Research Databases |
|  |  | Search modes - Boolean/Phrase | Search Screen - Advanced Search |
|  |  |  | Database - MEDLINE |
