# Supplementary material for: Communicating with patients and families about illness progression and end of life: a review of studies using direct observation of clinical practice
Source: BMC Palliat Care. 2021 Dec 8;20:186. doi: 10.1186/s12904-021-00876-2 (PMC8651503; doi:10.1186/s12904-021-00876-2)
Supplement: Supplementary file 2 — Additional file 2: Supplementary file 2: Characteristics of included studies. [file 12904_2021_876_MOESM2_ESM.docx]

**Supplementary file 2: Characteristics of included studies**

| **Reference** | **Country** | **Data Characteristics:**  *Setting, sample, data type* | **Analytic Approach** | **Analytic Depth** |
| --- | --- | --- | --- | --- |
| Ahluwalia et al. (2014) ^59^ | USA | - Outpatient cardiology appointments at two medical centres - 71 appointments, 52 patients, 44 doctors, 25 advance care planning conversations - Audio-only recorded | Qualitative content analysis | Low (single statements analysed outside of sequence of production) |
| Anderson et al. (2020) ^44^ | UK | - Hospice: 37 relatives/friends of patients, 5 clinicians - 29 consultations - Audio-only recorded | Conversation analysis | High (sequential analysis of what is said and how it is said) |
| Anderson et al. (2020) ^48^ | UK | - Hospice: 32 relatives/friends of patients, 5 clinicians - 20 conversations - Audio-only recorded | Conversation analysis | High (sequential analysis of what is said and how it is said) |
| Chou et al. (2017) ^52^ | USA | - 2 cancer hospitals, 9 oncologists, 26 cancer patients - 26 appointments - Audio-visual recordings (but only transcripts were analysed) | Discourse analysis | Low (single statements analysed outside of sequence of production) |
| Cortez et al. (2019) ^53^ | USA | - Cancer hospitals: 4 hospitals, 51 triads comprising a doctor, patient, and caregiver - 128 visits - Audio-only recordings | Conversation analysis | High (sequential analysis of what is said and how it is said) |
| Ekberg et al. (2020) ^40^ | Australia | - Hospital paediatric palliative care: 8 families, precise number of family members and clinicians not reported - 9 consultations - Audio-visual recordings | Conversation analysis | High (sequential analysis of what is said and how it is said) |
| Ekberg et al. (2019) ^43^ | Australia | - Hospital paediatric palliative care: 8 families, precise number of family members and clinicians not reported - 9 consultations - Audio-visual recordings | Conversation analysis | High (sequential analysis of what is said and how it is said) |
| Ford et al. (2019) ^47^ | UK | - 1 hospice, 5 doctors, 37 patients, 17 companions - 37 conversations - Most audio-visual recorded, a few audio-only recordings | Conversation analysis | High (sequential analysis of what is said and how it is said) |
| Gill (2019) ^55^ | USA | - 1 breast clinic and 1 general surgery clinic (not clear which site study data comes from) - 1 consultation - Audio-only recording | Conversation analysis | High (sequential analysis of what is said and how it is said) |
| Land et al. (2019) ^46^ | UK | - 1 hospice, 5 consultants, 37 patients and their companions - 37 consultations - Mixture of audio-visual and audio-only recordings | Conversation analysis | High (sequential analysis of what is said and how it is said) |
| Lutfey & Maynard (1998) ^49^ | USA | - Oncology consultations:1 oncologist, 3 patients with terminal cancer and their partners. - 3 consultations - Audio-visual recordings | Conversation analysis | High (sequential analysis of what is said and how it is said) |
| Maynard et al. (2016) ^56^ | USA | - Cancer hospitals: 4 hospitals, 51 triads comprising a doctor, patient, and caregiver - 128 visits - Audio-only recordings | Conversation analysis | High (sequential analysis of what is said and how it is said) |
| Norton et al. (2013) ^45^ | USA | - Inpatient palliative care team consultations, 66 interactions, 13 doctors, 2 nurse practitioners - 66 consultations - Audio-only recordings | Qualitative analysis (analytic focus similar to conversation analysis and discourse analysis approaches) | High (sequential analysis of what is said and how it is said) |
| Pecanac (2017) ^57^ | USA | - Intensive care: 2 units, precise number of participants not reported - 36 family conference conversations - Audio-only recordings | Conversation analysis | High (sequential analysis of what is said and how it is said) |
| Peräkylä & Bor (1990) ^62^ | UK | - Counselling: HIV clinic sessions for both initial testing, and for patients previously tested positive. - Total sample not stated, 53 episodes analysed - Audio-visual recordings | Conversation analysis | High (sequential analysis of what is said and how it is said) |
| Peräkylä (1993) ^60^ | UK | - Counselling: 2 AIDS clinics, 7 counsellors, 20 patients, most patients accompanied by family members - 27 counselling interviews - Audio-visual recordings | Conversation analysis | High (sequential analysis of what is said and how it is said) |
| Peräkylä (1995) ^61^ | UK | - Counselling: Counselling: 2 AIDS clinics, 7 counsellors, 20 patients, most accompanied by family members - 27 counselling interviews - Audio-visual recordings | Conversation analysis | High (sequential analysis of what is said and how it is said) |
| Pino et al. (2016) ^42^ | UK | - 1 hospice, 5 doctors, 37 patients, 17 companions - 37 conversations - Most audio-visual recordings, a few audio-only recordings | Conversation analysis | High (sequential analysis of what is said and how it is said) |
| Pino & Parry (2019) ^41^ | UK | - 1 hospice, 5 doctors, 37 patients, 17 companions - 37 conversations - Most audio-visual recordings, a few audio-only recordings | Conversation analysis | High (sequential analysis of what is said and how it is said) |
| Rodriguez et al. (2007) ^50^ | Australia | - Oncology: 2 outpatient clinics, 6 oncologists, 29 patients with terminal metastatic cancer, most accompanied by family members - 175 episodes comprising single statements - Audio-only recordings | Content analysis | Low (single statements analysed outside of sequence of production) |
| Rodriguez et al. (2008) ^51^ | Australia | - Oncology: 2 outpatient clinics, 6 oncologists, 29 patients with terminal metastatic cancer, most accompanied by family members - 175 episodes comprising single statements - Audio-only recordings | Qualitative, constant comparison and coding | Low (single statements analysed outside of sequence of production) |
| Shaw at al. (2016) ^58^ | UK | - Neonatal intensive care, 1 unit, 6 consultants, 9 families - 16 conversations - Audio-only recording | Conversation analysis | High (sequential analysis of what is said and how it is said) |
| Shaw et al. (2017) ^64^ | Canada | - CALM therapy: 4 therapists, 10 patients with advanced cancer - 10 therapy sessions - Audio-only recording | Conversation analysis | High (sequential analysis of what is said and how it is said) |
| Shaw et al. (2019) ^65^ | Canada | - CALM therapy: 1 therapist, 1 patient with advanced cancer - 7 therapy sessions - Audio-only recordings | Conversation analysis | High (sequential analysis of what is said and how it is said) |
| Silverman & Peräkylä (1990) ^63^ | UK | - Counselling: 4 AIDS clinics. - Total sample not stated. 100 episodes analysed - Mixture of audio-visual and audio-only recordings | Conversation analysis | High (sequential analysis of what is said and how it is said) |
| Tate (2020) ^54^ | USA? | - Oncology: Cancer clinics, 2 oncologists, 82 patients - 90 consultations - Audio-visual recordings | Conversation analysis | High (sequential analysis of what is said and how it is said) |
