# Supplementary material for: Communicating with patients and families about illness progression and end of life: a review of studies using direct observation of clinical practice
Source: BMC Palliat Care. 2021 Dec 8;20:186. doi: 10.1186/s12904-021-00876-2 (PMC8651503; doi:10.1186/s12904-021-00876-2)
Supplement: Supplementary file 1 [file 12904_2021_876_MOESM2_ESM.docx]

**Supplementary file 2: Characteristics of included studies**

| **Reference** | **Country** | **Data Characteristics:** *Setting, sample, data type* | **Analytic Approach** | **Analytic Depth** |
| --- | --- | --- | --- | --- |
| Anderson et al. (2020) ^44^ | UK | Hospice: 37 relatives/friends of patients, 5 clinicians 29 consultations Audio-only recorded | Conversation analysis | High (sequential analysis of what is said and how it is said) |
| Anderson et al. (2020) ^48^ | UK | Hospice: 32 relatives/friends of patients, 5 clinicians 20 conversations Audio-only recorded | Conversation analysis | High (sequential analysis of what is said and how it is said) |
| Chou et al. (2017)^ 52^ | USA | 2 cancer hospitals, 9 oncologists, 26 cancer patients 26 appointments Audio-visual recordings (but only transcripts were analysed) | Discourse analysis | Low (single statements analysed outside of sequence of production) |
| Cortez et al. (2019) ^53^ | USA | Cancer hospitals: 4 hospitals, 51 triads comprising a doctor, patient, and caregiver 128 visits Audio-only recordings | Conversation analysis | High (sequential analysis of what is said and how it is said) |
| Ekberg et al. (2020) ^40^ | Australia | Hospital paediatric palliative care: 8 families, precise number of family members and clinicians not reported 9 consultations Audio-visual recordings | Conversation analysis | High (sequential analysis of what is said and how it is said) |
| Ekberg et al. (2019) ^43^ | Australia | Hospital paediatric palliative care: 8 families, precise number of family members and clinicians not reported 9 consultations Audio-visual recordings | Conversation analysis | High (sequential analysis of what is said and how it is said) |
| Ford et al. (2019) ^47^ | UK | 1 hospice, 5 doctors, 37 patients, 17 companions 37 conversations Most audio-visual recorded, a few audio-only recordings | Conversation analysis | High (sequential analysis of what is said and how it is said) |
| Gill (2019) ^55^ | USA | 1 breast clinic and 1 general surgery clinic (not clear which site study data comes from) 1 consultation Audio-only recording | Conversation analysis | High (sequential analysis of what is said and how it is said) |
| Land et al. (2019) ^46^ | UK | 1 hospice, 5 consultants, 37 patients and their companions 37 consultations Mixture of audio-visual and audio-only recordings | Conversation analysis | High (sequential analysis of what is said and how it is said) |
| Lutfey & Maynard (1998) ^49^ | USA | Oncology consultations:1 oncologist, 3 patients with terminal cancer and their partners. 3 consultations Audio-visual recordings | Conversation analysis | High (sequential analysis of what is said and how it is said) |
| Maynard et al. (2016) ^56^ | USA | Cancer hospitals: 4 hospitals, 51 triads comprising a doctor, patient, and caregiver 128 visits Audio-only recordings | Conversation analysis | High (sequential analysis of what is said and how it is said) |
| Norton et al. (2013) ^45^ | USA | Inpatient palliative care team consultations, 66 interactions, 13 doctors, 2 nurse practitioners 66 consultations Audio-only recordings | Qualitative analysis (analytic focus similar to conversation analysis and discourse analysis approaches) | High (sequential analysis of what is said and how it is said) |
| Pecanac (2017) ^57^ | USA | Intensive care: 2 units, precise number of participants not reported 36 family conference conversations Audio-only recordings | Conversation analysis | High (sequential analysis of what is said and how it is said) |
| Peräkylä & Bor (1990) ^62^ | UK | Counselling: HIV clinic sessions for both initial testing, and for patients previously tested positive. Total sample not stated, 53 episodes analysed Audio-visual recordings | Conversation analysis | High (sequential analysis of what is said and how it is said) |
| Peräkylä (1993) ^60^ | UK | Counselling: 2 AIDS clinics, 7 counsellors, 20 patients, most patients accompanied by family members 27 counselling interviews Audio-visual recordings | Conversation analysis | High (sequential analysis of what is said and how it is said) |
| Peräkylä (1995) ^61^ | UK | Counselling: Counselling: 2 AIDS clinics, 7 counsellors, 20 patients, most accompanied by family members 27 counselling interviews Audio-visual recordings | Conversation analysis | High (sequential analysis of what is said and how it is said) |
| Pino et al. (2016) ^42^ | UK | 1 hospice, 5 doctors, 37 patients, 17 companions 37 conversations Most audio-visual recordings, a few audio-only recordings | Conversation analysis | High (sequential analysis of what is said and how it is said) |
| Pino & Parry (2019) ^41^ | UK | 1 hospice, 5 doctors, 37 patients, 17 companions 37 conversations Most audio-visual recordings, a few audio-only recordings | Conversation analysis | High (sequential analysis of what is said and how it is said) |
| Rodriguez et al. (2007) ^50^ | Australia | Oncology: 2 outpatient clinics, 6 oncologists, 29 patients with terminal metastatic cancer, most accompanied by family members 175 episodes comprising single statements Audio-only recordings | Content analysis | Low (single statements analysed outside of sequence of production) |
| Rodriguez et al. (2008) ^51^ | Australia | Oncology: 2 outpatient clinics, 6 oncologists, 29 patients with terminal metastatic cancer, most accompanied by family members 175 episodes comprising single statements Audio-only recordings | Qualitative, constant comparison and coding | Low (single statements analysed outside of sequence of production) |
| Shaw at al. (2016) ^58^ | UK | Neonatal intensive care, 1 unit, 6 consultants, 9 families 16 conversations Audio-only recording | Conversation analysis | High (sequential analysis of what is said and how it is said) |
| Shaw et al. (2017) ^64^ | Canada | CALM therapy: 4 therapists, 10 patients with advanced cancer 10 therapy sessions Audio-only recording | Conversation analysis | High (sequential analysis of what is said and how it is said) |
| Shaw et al. (2019) ^65^ | Canada | CALM therapy: 1 therapist, 1 patient with advanced cancer 7 therapy sessions Audio-only recordings | Conversation analysis | High (sequential analysis of what is said and how it is said) |
| Silverman & Peräkylä (1990) ^63^ | UK | Counselling: 4 AIDS clinics. Total sample not stated. 100 episodes analysed Mixture of audio-visual and audio-only recordings | Conversation analysis | High (sequential analysis of what is said and how it is said) |
| Tate (2020) ^54^ | USA? | Oncology: Cancer clinics, 2 oncologists, 82 patients 90 consultations Audio-visual recordings | Conversation analysis | High (sequential analysis of what is said and how it is said) |
